# Acknowledgement of manuscript reviewers 2015

**DOI:** 10.1186/s13722-016-0054-9

**Published:** 2016-03-21

**Authors:** Jeffrey H. Samet

**Affiliations:** Boston University School of Medicine, Boston, MA USA

## Contributing reviewers

The editor of *Addiction Science & Clinical Practice* would like to thank all our reviewers who have contributed to the journal in Volume 10 (2015).

**Janette Baird**

USA

**Edward Bernstein**

USA

**Daniel Blonigen**

USA

**Marcel Bonn-Miller**

USA

**Janice Brown**

USA

**Aaron Fox**

USA

**Daniel Fuster**

Spain

**Deborah Garnick**

USA

**Lillian Gelberg**

USA

**Carolyn Greene**

USA

**Mark Greenwald**

USA

**Doan Ha**

USA

**Hildi Hagedorn**

USA

**Kim Hamlett-Berry**

USA

**Dana Hancock**

USA

**Henrick Harwood**

USA

**Nancy Haug**

USA

**Lon Hays**

USA

**Heidi Hutton**

USA

**Hussam Jefee-Bahloul**

USA

**Aaron Johnson**

USA

**Nestor Damian Kapusta**

Austria

**Theresa Kim**

USA

**Jan Klimas**

Ireland

**Keren Lehavot**

USA

**Verena Metz**

USA

**John Muench**

USA

**Amy O’Donnell**

UK

**E. Roberto Orellana**

USA

**Einat Peles**

Israel

**Michael Prendergast**

USA

**Gerda M. Saletu-Zyhlarz**

Austria

**Eric Schmidt**

USA

**Christopher Shanahan**

USA

**Frances Stillman**

USA

**Jeanette Tetrault**

USA

**Marta Torrens**

Spain

**Anne Unger**

Austria

**Sarah Wakeman**

USA

**Michael Weaver**

USA

**Melissa Weimer**

USA

**Kenneth Weingardt**

USA

